# Clinical, genetic, and experimental research of hyperphenylalaninemia

**DOI:** 10.3389/fgene.2022.1051153

**Published:** 2023-01-04

**Authors:** Anqi Chen, Yukun Pan, Jinzhong Chen

**Affiliations:** ^1^ Department of Forensic Medicine, School of Basic Medical Sciences, Shanghai Medical College, Fudan University, Shanghai, China; ^2^ Barbell Therapeutics Co. Ltd., Shanghai, China; ^3^ State Key Laboratory of Genetic Engineering, Institute of Genetics, School of Life Sciences, Fudan University, Shanghai, China

**Keywords:** phenylalanine (Phe), hyperphenylalaninemia (HPA), phenylketonuria (PKU), phenylalanine hydroxylase (PAH), tetrahydrobiopterin (BH4), DnaJ heat shock protein family (Hsp40) member C12 (DNAJC12), dietary restriction, gene therapy

## Abstract

Hyperphenylalaninemia (HPA) is the most common amino acid metabolism defect in humans. It is an autosomal-recessive disorder of the phenylalanine (Phe) metabolism, in which high Phe concentrations and low tyrosine (Tyr) concentrations in the blood cause phenylketonuria (PKU), brain dysfunction, light pigmentation and musty odor. Newborn screening data of HPA have revealed that the prevalence varies worldwide, with an average of 1:10,000. Most cases of HPA result from phenylalanine hydroxylase (PAH) deficiency, while a small number of HPA are caused by defects in the tetrahydrobiopterin (BH4) metabolism and DnaJ heat shock protein family (Hsp40) member C12 (DNAJC12) deficiency. Currently, the molecular pathophysiology of the neuropathology associated with HPA remains incompletely understood. Dietary restriction of Phe has been highly successful, although outcomes are still suboptimal and patients find it difficult to adhere to the treatment. Pharmacological treatments, such as BH4 and phenylalanine ammonia lyase, are available. Gene therapy for HPA is still in development.

## Introduction

Phenylketonuria (PKU), the severe form of hyperphenylalaninemia (HPA), has been reported for near 90 years (Fölling. 1934; Grisch-Chan et al., 2019). It is the most common metabolic disorder of amino acid metabolism in humans, which is recognized by accumulated phenylalanine (Phe) in blood with an average incidence of 1:10,000 of the populations (Hillert et al., 2020). Although extensive works have been carried out to elucidate the pathological mechanism, the approach to HPA treatment has been rarely updated. Since the 1950s, a Phe-restricted diet has been the standard treatment for control the high blood Phe levels found in HPA (Sarkissian et al., 1999). Dietary management as a therapy remains a reliable and effective approach for preventing the manifestations of HPA for many (MacDonald et al., 2020). Recently, two drugs have been approved and used successfully for the correction of HPA (Keil et al., 2013; Burton et al., 2020). Several genetic diseases have been relieved using gene therapies (Welch et al., 2007; Sun, Zheng, and Simeonov 2017; Pogue et al., 2018), but the complete non-dietary treatment for HPA stays at very early-stage. This review aims to summarize the current understanding of HPA from the aspects of history, dietary treatments, pharmacological approaches, as well as the ongoing experimental gene therapies.

## Milestones in PKU

In 1934, doctor Fölling (Fölling, 1934) studied two siblings with an intellectual disability and a musty odor and identified phenylpyruvic acid in their urine. The disease was named Følling’s disease and described as the inheritance of phenylpyruvic amentia (phenylketonuria, PKU) (Penrose 1935). PKU was the result of impaired Phe to Tyr conversion (Jervis 1947). Twenty years after Følling’s report, the first description of a Pherestricted diet began a new era of PKU therapy (Bickel, Gerrard, and Hickmans 1953). In 1963, the Guthrie bacterial inhibition assay (Scriver 1998) was established for detecting PKU in newborns. In 1971, the catalytic characteristics of the PAH system were determined (Kaufman 1971). A second cause of HPA, a defect in the tetrahydrobiopterin (BH4) metabolism, was identified in 1974 (Bartholome 1974). The third cause of HPA, a DnaJ heat shock protein family (Hsp40) member C12 (DNAJC12) variant, was identified in 2017 (Anikster et al., 2017). Regarding pharmacological treatment, BH4, as an adjunct treatment, decreases blood Phe concentrations in some cases (Kure et al., 1999) and phenylalanine ammonia lyase (PAL) provides an alternate treatment option (Longo et al., 2014).

## Epidemiology

More than 50 years ago, measurement of the blood Phe concentrations in newborns was developed. Since then, this approach has been widely used worldwide (Guthrie and Susi 1963). Normal Phe concentrations are below 120 μmol/L, and Phe/Tyr is below 1.5. The prevalence of HPA varies worldwide (van Spronsen et al., 2021). In Europe, the prevalence ranges from 1:2,700 live births in Italy to <1:100,000 live births in Finland (Hillert et al., 2020). HPA prevalence is the lowest in Asian populations, such as Thailand, where it is 1:212,535 (Sutivijit, Banpavichit, and Wiwanitkit 2011). In China, the prevalence is 1:159,24 live births and ranges from 1:349,63 (Gansu Province) to 1: 666,667 (Guangxi Municipality) live births (Xiang et al., 2019).

## Phe hydroxylation pathway and HPA

PAH, chaperone DNAJC12, and the BH4 system are necessary for metabolizing Phe to Tyr. Pathogenetic mutations in the genes synthesizing these enzymes are the potential causes of HPA, accumulating phenylpyruvate and hypotyrosinemia. The side way product phenylpyruvate is rapidly excreted in urine, but the tissue concentrations are probably too low to be of any clinical consequences (Jervis 1952). Hypotyrosinemia might be present in PKU cases and related to light pigmentation. However, there might be enough Tyr in food to support the Tyr-related neurotransmitter. Actually, supplementing the diet with Tyr does not prevent severe cognitive disability in individuals with PKU (Batshaw, Valle, and Bessman 1981). Restriction of Phe intake can prevent the major manifestations of PKU, which suggests HPA itself as the primary neurotoxin in PKU (van Vliet et al., 2018).

## Cerebral effects of HPA

**FIGURE 1:**
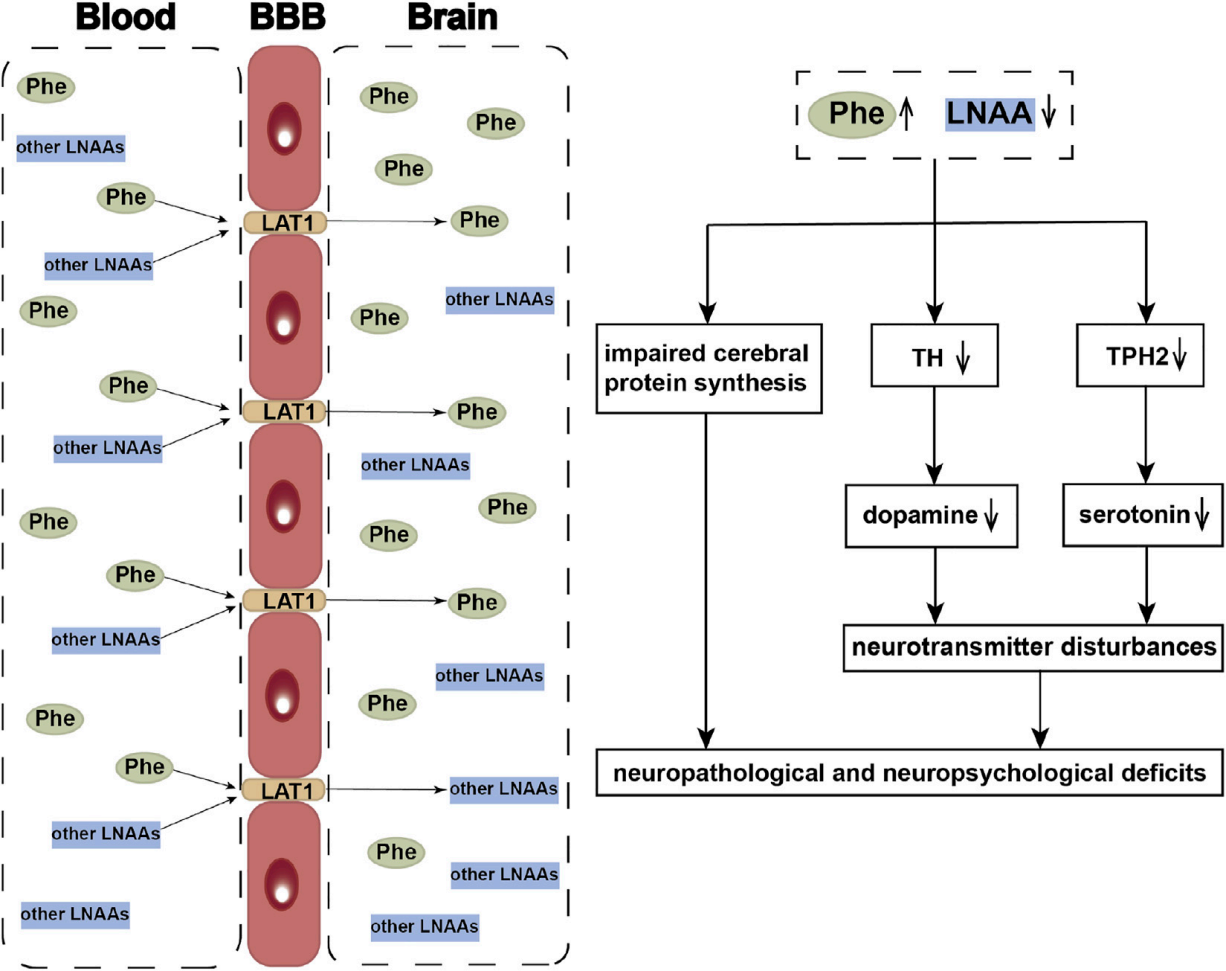
Neuropathological and neuropsychological deficits of HPA. LAT1 functions as a transporter mediating uptake of large neutral amino acids (LNAAs), such as phenylalanine (Phe), tyrosine (Tyr), and tryptophan *etc.*, across the blood-brain barrier (BBB). When phenylalanine hydroxylase is defective, the elevated blood Phe concentrations competitively inhibit the other LNAAs transporting into the brain. The reduced LNAAs concentrations contribute to the impaired cerebral protein synthesis and neurotransmitter deficiency, which further resulted in the occurrence of neuropathological and neuropsychological deficits. LNAAs, large neutral amino acids; Phe, phenylalanine; Tyr, tyrosine; TH, Tyr hydroxylase; TPH2, tryptophan hydroxylase 2.

The most severe manifestations in PKU patients are intellectual disability and epilepsy. Similar to other aromatic amino acids and other large neutral amino acids (LNAAs), Phe is transferred to the brain by the large neutral amino acid transporter 1 (LAT1) on the blood–brain barrier (BBB) (Kanai et al., 1998). High levels of Phe mediate competition for binding to LAT1 with other LNAAs, leading to their deficiency in the brain (de Groot et al., 2010). These deficiencies are probably responsible for the impaired cerebral protein synthesis in PKU (Hoeksma et al., 2009) and contribute to brain monoamine neurotransmitter deficiencies (van Vliet et al., 2015). High Phe levels in the brain can inhibit Tyr hydroxylase (TH) and tryptophan hydroxylase 2 (TPH2), the rate-limiting steps in dopamine and serotonin synthesis, which result in disturbances of the monoamine neurotransmitters implicated as contributors to the neuropsychiatric symptoms (de Groot et al., 2010). Supplementation of LNAAs without Phe has been promoted as a treatment approach to PKU, by competitive inhibition of Phe across the BBB (van Vliet et al., 2016) (Figure 1).

In vitro experiments with both cultured neurons and animal models, HPA caused disturbances of neuronal dendritic growth and synaptic connectivity (Hartwig et al., 2006; Hörster et al., 2006). HPA can impair the cholesterol or other brain lipids synthesis and thereby interfere with myelin-related structures (Shefer et al., 2000). HPA has also resulted in the forming of amyloid-like fibrils, a pathological structure similar to the amyloid plaques associated with Alzheimer’s disease (Adler-Abramovich et al., 2012).

The cerebral energy metabolism was abnormal in a PKU animal model. Glucose metabolism is reduced in the frontal cortex of hyperphenylalaninaemic Pah^enu2^ mice, which might be related to behavioral abnormalities (Qin and Smith 2007; Winn et al., 2016). HPA can inhibit pyruvate kinase or other enzymes involved in glycolysis or oxidative phosphorylation (Miller, Hawkins, and Veech 1973). Recently, several reports have suggested that HPA alters the methylation pattern and increases oxidative stress (Dobrowolski et al., 2016; van der Goot et al., 2019).

## Diagnosis and screening

The genotypes of patients with HPA are considered deficient in PAH, BH4 and DNAJC12 (Table 1). Most cases of HPA are caused by pathogenetic mutations of the PAH gene located on human chromosome 12 (Woo et al., 1983). More than 1000 PAH pathogenetic mutations have been reported where patients might be compound heterozygous for two different PAH variants, and more than 2000 genotypes resulted in the HPA phenotype (Hillert et al., 2020).

**TABLE 1:** The genes associated with hyperphenylalaninemia (HPA).

Deficiency	Gene	Inheritance	Location	Genomic coordinates (GRCh38)	MIM number
PAH deficiency	PAH	autosomal recessive	12q23.2	12:102,836,889–102,958,441	261600
BH4 deficiency	PTS	autosomal recessive	11q23.1	11:112,226,428–112,233,973	261640
	GCH1	autosomal recessive	14q22.2	14:54,842,017–54,902,826	233910
	QDPR	autosomal recessive	4p15.32	4:17,486,395–17,512,090	261630
	PCBD1	autosomal recessive	10q22.1	10:70,882,280–70,888,565	264070
DNAJC12 deficiency	DNAJC12	autosomal recessive	10q21.3	10:67,796,669–67,838,188	617384

PAH, phenylalanine hydroxylase; BH4, tetrahydrobiopterin; DNAJC12: DnaJ heat shock protein family (Hsp40) member C12; PTS: 6-pyruvoyltetrahydropterin synthase; GCH1: GTP, cyclohydrolase 1; QDPR: quinoid dihydropteridine reductase; PCBD1: pterin-4, alpha-carbinolamine dehydratase 1.

PAH pathogenetic mutations are inherited in an autosomal-recessive manner and result in expressing mutant protein with low/no catalytic activity, or even the absence of PAH protein expression (Garbade et al., 2019). BH4 deficiency due to inherited defects was found in the biopterin system, which consists of GTP cyclohydrolase 1 (GTPCH), 6-pyruvoyl-tetrahydropterin synthase (PTPS), dihydropteridine reductase (DHPR) or pterin-4a-carbinolamine dehydratase (PCD). PAH is disrupted in the absence of the chaperone DNAJC12, and this is described as an additional cause of inherited HPA (van Spronsen et al., 2017).

The Guthrie filter paper based newborn screening test for HPA has resulted in diagnoses in the neonatal period worldwide. To screen for PKU, the bacterial inhibition assay (BIA) and fluorimetric microassay (FMA) are employed to quantify Phe levels. A better method, tandem mass spectrometry (TMS) allows Phe and Tyr to be measured simultaneously. The PKU-positive screening result is determined by a cut-off of Phe concentrations ranging from 120 to 240 μmol/L in combination with a Phe to Tyr ratio >1.5–2 (van Wegberg et al., 2017).

Once HPA has been determined, it is necessary to distinguish PAH deficiency, disorders of the BH4 metabolism, and DNAJC12 defects. A panel comprising all genes known to cause HPA (possibly a larger panel with more than HPA-related genes) can provide final diagnostic confirmation and predict the metabolic phenotype in PAH deficiency. This is valuable for HPA diagnosis and treatment, especial for autosomal-recessive guanosine triphosphate cyclohydrolase (GTPCH) or serine racemase (SR) deficiency, which might present with normal blood Phe in the neonatal screening (van Wegberg et al., 2017). Thanks to the improvement of genotyping, most BH4 loading tests are covered for BH4 deficiencies, even in all HPA cases (Blau et al., 2014).

## Dietary management

HPA screening aims for early diagnosis and prevention of intellectual disability by dietary management. Since the intellectual disability caused by PKU is irreversible, the prevention is more important than treatment. The dietary management of PKU should be initiated as soon as possible to prevent the cognitive and neurologic deficits (Evers et al., 2020). Unlike patients who experienced the neurocognitive consequences of late diagnosis and treatment, patients diagnosed and treated as infants may experience improved growth and development (Knox 1960). For late-diagnosed PKU patients, dietary management is also recommended, because behavior and epilepsy can be improved after dietary control (Koch et al., 1999).

Phe is an essential amino acid that cannot be produced by the body, and the blood Phe level is highly dependent on Phe intake. For this reason, dietary management should be successful. However, there is still a higher incidence of attention-deficit–hyperactivity disorder and specific learning disabilities in PKU, even with good dietary control (Arnold et al., 2004). Dietary management is comprised of limited natural protein intake, supplementation with a Phe-free amino acid mixture, and consumption of low-protein food products (MacDonald et al., 2020). Although the dietary treatment must be individualized and monitored, the protein and Phe deficiency can also cause adverse effects such as growth restriction, anorexia, alopecia, lethargy, and eczematous eruptions (Hanley et al., 1970). The basis of dietary treatment has changed little since 1953. Despite substantial efforts to improve quality, taste, and consumption methods of Phe-free amino acid mixtures, acceptance can be poor (Daly et al., 2021). Phe control during childhood is particularly important. The children themselves, as well as their family members, should understand the importance of Phe control, and sometimes it is necessary to hold the method of Phe test (Bilginsoy et al., 2005). Higher blood Phe concentrations are not always associated with poorer neurocognitive outcomes (Leuzzi et al., 2020), and the need to decrease blood Phe levels in adult PKU patients is unsure (Burlina et al., 2019). These adult patients might consider stopping dietary treatment. Consequently, they may not resume normal natural protein intake and be at risk of deficiency for some micronutrients (Lammardo et al., 2013). Regarding maternal PKU, the risk of fetal developmental abnormalities is increased if the maternal blood Phe concentrations exceed 360 μmol/L. For optimal metabolic control, the American College of Medical Genetics (ACMG) recommends lifelong maintenance of Phe concentrations within the range of 120–360 μmol/L (van Wegberg et al., 2017).

## Pharmacological treatments

Sapropterin (Kuvan^®^, BioMarin) is a BH4 synthetic analog, an oral drug approved by FDA in 2008. Sapropterin is an exogenous synthetic BH4, and it is given as an effective replacement for endogenous BH4. The rationale for Sapropterin administration is to restore the Phe metabolism by enhancing the activity of the defective PAH. Since BH4 is a cofactor of PAH, the excess cofactor would help stimulate residual PAH to process Phe, and thereby decrease the blood Phe concentrations (Battaglia-Hsu and Feillet, 2010; Dubois and Cohen 2010). Sapropterin can increase dietary Phe tolerance (Keil et al., 2013) in some patients, thus enabling them to liberalize dietary restrictions. However, this drug does not response to all patients with PKU or BH4 deficiency (Dubois and Cohen 2010). Ten years later, a pegylated Phe ammonia lyase (pPAL, Palynziq^®^, BioMarin), was approved by FDA. The rationale for Palynziq^®^ to reduce blood Phe concentrations through converting Phe to ammonia and transcinnamic acid (Thomas et al., 2018). The drug also changed the lives of PKU patients (Burton et al., 2020). Based on clinical experience and knowledge of adverse immunological events, the guidelines for pegvaliase treatment induction and maintenance in PKU patients have been proposed (Longo et al., 2019). Of all patients, 60.7% were able to achieve blood Phe concentrations less than 360 μmol/L without restricting dietary protein intake (Thomas et al., 2018). The pPAL injection for treatment of PKU patients has proved effective (Thomas et al., 2018). As a foreign protein, the immune-mediated responses remain the most important safety issue for this drug (Gupta et al., 2018).

## Experimental therapies

The lifelong restricted diet supplemented with Phe-free protein substitutes has been the gold standard treatment for PKU patients (Burlina et al., 2020). However, the patient adherence to this therapy trends to be difficult after childhood, owing to the substantial time, cost and lifestyle burdens (Rose et al., 2019). Consequently, an effective, non-pathological, and long-term non-dietary restriction treatment is urgently needed. Gene therapy has been applied to cure genetic diseases such as spinal muscular atrophy (SMA) (Mendell et al., 2017; Waldrop et al., 2020) and hemophilia B (Lu et al., 2016; George et al., 2017). New approaches using gene therapy for PKU are considered promising because of the results from established PKU murine and mouse models (Isabella et al., 2018; Li et al., 2021). US and European authorities have approved injectable pegvaliase for PKU, at the same time, they have also incited other approaches to improve enzyme-based therapies to decrease the frequency and severity of this adverse effect (Hydery and Coppenrath 2019).

Rubius Therapeutics evaluated the safety and tolerability of RTX-134, the allogeneic human red blood cells (RBCs) expressing the AvPAL (which consists an Anabaena variabilis phenylalanine ammonia lyase gene inside the cell); unfortunately, this clinical trial failed to provide the expected signals of efficacy. Although the clinical trial using liver cell transplantation is in the recruiting process, the potential for transplant rejection will be unavoidable. Therefore, all participants must be treated with life-long immune suppression medications (NCT01465100), which will expose the patients to higher risks of an immune-mediated adverse reaction. Compared to the transplantation of enzyme-loaded RBCs, the enzyme substitution therapy with phenylalanine ammonialyase (PAL) appears more promising for decreasing Phe concentrations. CDX-6114 (Codexis Inc. And Nestlé) is a PAL-like enzyme that can remove Phe during digestion. To date, the results from three clinical trials (NCT03577886, NCT03797664, and NCT04085666) showed that the drug was well tolerated at different dose levels without any serious adverse events or GI-related symptoms. Unfortunately, further investigation was stopped because of the altered compositions. Other orally administered enzymes are SYNB 1618 and SYNB 1943 (Synlogic Biotic), which are engineered bacterial therapeutic drugs for oral delivery. SYNB 1618 and SYNB 1943 are expected to control Phe levels in patients with PKU. The strong positive results with these two drugs will undoubtedly initiate a phase 3 clinical trial in the near future. It has been reported that more than 10% catalytic activity is necessary for correcting PHA to below 700 μmol/L Phe (Hamman et al., 2005). However, the results observed from several PKU-related gene therapies were far from satisfactory, which suggested the treatments were still in an early stage.

For genetic disorders, genome editing using recombinant adeno-associated virus (rAAV) vectors is one of the most popular gene therapy strategies (Wang, Tai, and Gao 2019). The open read frame (ORF) of PAH is relatively small, which makes it fit well to the AAV vector. Furthermore, the native expression and residence of PAH is in liver cells, where the protein expression is easy and intracellular proteins would not be exposed to the immune system (Wang, Tai, and Gao 2019). The benefits mentioned above have laid the foundations for AAV-based gene therapy for PKU.

Since PKU is a genetic disorder owing to the mutation in the PAH gene, genome editing-based gene therapies have been developed to modify the genes. AAV-based CRISPR-Cas base editors provided more than 20% PAH activity in Pah^enu2^ mice and restored physiological blood Phe concentrations (Villiger et al., 2018; Richards et al., 2020). By using a dual AAV-based editing system, Zhou et al. (Zhou et al., 2022) achieved efficient correction of PKU-related mutations *in vitro* and *in vivo*. Co-delivery of SaCas9/sgRNA/donor templates with AAV receptor (AAVR) *via* AAV8 vectors also corrected the single mutation (PahR408W), and dramatically increased the editing efficiency *in vivo* (Yin et al., 2022). The advantage of this method is that the modified cells were maintained throughout the hepatocyte proliferation. Although effective in mice, no human clinical trials are reported. In addition, AAV-based gene therapies with AAV2/5-PAH were not successful; they could not correct the PAH deficiency without a high dosage of AAV (1014 vg/mouse) that might result in liver damage (Mochizuki et al., 2004; Oh et al., 2004). Alternatively, vectors pseudotyped with capsids from AAV serotype 8 were generated to explore a long-term non-dietary restriction treatment. The rAAV serotype 8 vectors exerted neither hepatic toxicity nor immunogenicity in the treated mice, and the blood Phe decreased to normal levels independent of the gender differences (Ding, Georgiev, and Thöny 2006; Rebuffat et al., 2010; Tao et al., 2020). At the same time, therapeutic ranges of Phe reverted the hair from brown to black in PAH-deficient mice. The better results came from AAV2/8-PAH (Rebuffat et al., 2010) and AAVHSC15 (Ahmed et al., 2020), which obtained a long-lasting correction of PAH activity in Pah^enu2^ mice. Similar observations were made using either the recombinant triplecistronic AAV2 pseudotype 1 vector (Ding et al., 2008) or the pseudotyped rAAV2/8-hPAH vector (Jung et al., 2008). Remarkably, the offspring of the treated mice were rescued from the pathologic effect (Jung et al., 2008). In a comparative study, both the rAAV2 pseudotype 1 (rAAV2/1) and rAAV2/8 vectors showed the long-term phenylalanine clearance. Although an elevated phenylalanine level was detected in female mice after 8–10 months of rAAV2/8 injection, it was corrected by either administering synthetic tetrahydrobiopterin supplementation or injecting a different AAV pseudotype vector (Rebuffat et al., 2010). Overall, the rAAV8 vectors not only corrected hyperphenylalaninemia in both males and females, but, more importantly, they exerted neither hepatic toxicity nor immunogenicity in Phe-deficient mice.

The effective outcomes and the feasibility of a single intravenous injection have paved the way to develop the clinical gene therapy procedure for PKU patients. At present, two activated clinical trials are ongoing with AAVHSC15 (NCT03952156 and NCT05222178), and one with AAV2/8 (NCT04480567). HMI-102 and HMI-103 exerted therapeutic effects by using an AAVHSC15 vector containing a functional copy of the human PAH gene. Both of them were the *in vivo* treatments that delivered functioning PAH genes to the liver by one-time I.V. administration. The difference is in the way the drugs work in the body. HMI-102 is designed to deliver the functional gene in the form of episomes, and HMI-103 creates the functional PAH protein by the integrated PAH gene and unintegrated PAH episomes (Table 2).

**TABLE 2:** Summary of the clinical trials and AAV-based experimental gene therapies.

Clinical trials								
Study title	Interventions	Delivery	Phase	ClinicalTrials.gov identifier	Sponsor and principal investigator	Start date	Completion date (estimated)	Ages eligible for study
AAV Gene Therapy Study for Subjects With PKU	BMN 307: AAV2/8 gene therapy infusion	Systemic delivery	Phase 1/2 Active, not recruiting	NCT04480567	BioMarin Pharmaceutical	24 September 2020	December 2027	15 Years and older
Gene Therapy Clinical Study in Adult PKU (pheNIX)	HMI-102: AAVHSC15 vector expressing human PAH (HMI-102)	Systemic delivery	Phase 1/2: Recruiting	NCT03952156	Homology Medicines, Inc.	10 June 2019	September 2023	18 Years–55 Years
Safety and Efficacy of HMI-103, a Gene Editing Development Candidate in Adults with Classical PKU Due to PAH Deficiency	HMI-103: an AAVHSC15 vector HMI-103 Phe EDIT	Systemic delivery	Phase 1: Recruiting	NCT05222178	Homology Medicines, Inc.	3 June 2022	June 2028	18 Years–55 Years
Liver Cell Transplant for Phenylketonuria	Hepatocyte transplantation	Transplantation	Phase 1/2: Recruiting	NCT01465100	Ira Fox	15 December 2022	December 2023	14 Years–55 Years
Safety and Tolerability of RTX-134 in Adults with Phenylketonuria	RTX-134 RBC	Systemic delivery	Phase I: failure	NCT04110496	Rubius	29 January 2020	March 2035	18 Years and older
Safety and Tolerability of CDX-6114 in Healthy Volunteers	CDX-6114	Oral delivery	Phase 1: completed	NCT03577886	Codexis Inc	4 July 2018	4 September 2018	18 Years–55 Years
A Study of the Safety and Tolerability of CDX-6114 in Healthy Volunteers			Phase 1: completed	NCT03797664	Codexis Inc	14 December 2018	12 April 2019	18 Years–55 Years
Pharmacodynamics, Safety, Tolerability and Pharmacokinetics of CDX-6114 in Patients with Phenylketonuria (PKU)			Phase 1: completed	NCT04085666	Nestlé	1 June 2019	30 August 2020	18 Years–55 Years
Safety, Tolerability, Pharmacodynamics and Pharmacokinetics of CDX 6114 in PKU Patients			Withdrawn (study product composition to move from liquid to solid)	NCT04256655	Nestlé	1 December 2020	30 December 2021	18 Years–65 Years
Safety and Tolerability of SYNB1618 in Healthy Adult Volunteers and Adult Subjects With Phenylketonuria (PKU)	SYNB1618: engineered bacterial drug overexpressing PAL to metabolize phenylalanine in the gut	Oral delivery	Phase 1/2a: completed	NCT03516487	Synlogic	17 April 2018	21 June 2019	18 Years–64 Years
Safety and Tolerability of SYNB1934 in Healthy Adult Volunteers	SYNB1618 and 1943: engineered bacterial drug overexpressing PAL to metabolize phenylalanine in the gut		Phase 1: completed	NCT04984525		2 July 2021	10 December 2021	18 Years–64 Years
Efficacy and Safety of SYNB1618 and SYNB1934 in Adult Patients With Phenylketonuria (SynPheny-1)			Phase 2: recruiting	NCT04534842		25 August 2020	August 2022	18 Years and older

Experimental therapies					
Vectors	Treatment	Delivery	Insightful Conclusions	Investigator	References
liver-tropic rAAV2/8 vectors	A liver-tropic rAAV2/8 vectors + Vanillin Treatment	Systemic delivery	• Lifelong and permanent correction of the Pah^enu2^ allele in a portion of treated hepatocytes	Department of Molecular and Medical Genetics, Oregon Health & Science	Mol Ther Methods Clin Dev. 2019 December 24; 17:234–245
			• Partial restoration of liver PAH activity		
			• Substantial reduction of blood Phe		
			• Prevention of maternal PKU effects during breeding		
AAV-base editor systems (CRISPR-Cas-associated base editors)	An rAAV vectors CRISPR-Cas-associated base editors	Systemic delivery	• Restored physiological Phe blood levels	Department Biology, Institute for Molecular Health Sciences, ETH Zürich	Nat Med. 2018 October; 24 (10):1519–1525
			• Up to 63% mRNA correction rates		
			• Restoration of 65% PAH enzyme activity		
			• Reversion of hypopigmented phenotype		
AAV-Anc80	The synthetic AAV vector Anc80 *via* systemic administration to deliver a functional copy of a codon-optimized human PAH	Systemic delivery	• A safely and durably PKU curation	Children’s Hospitals and Clinics of Minnesota, Minneapolis, Minnesota, United States.	J Inherit Metab Dis. 2021 November; 44 (6):1369–1381
			• The significant and durable reduction of circulating Phe (nearly decreased to control levels in males)		
			• Reversion of hypopigmented phenotype		
AAV8-PAL	An rAAV8 viral vector expressing the humanized PAL under the control of human antitrypsin (hAAT) promoter	Systemic delivery	• Long-term HPA correction in both genders	State Key Laboratory of Biotherapy and Cancer Center, West China Hospital, Sichuan University, Chengdu, Sichuan 610041, China	Mol Ther Methods Clin Dev. 2020 January 13; 19:507–517
AAV2-PKU-5 and rAAV2-PKU-5/8	An rAAV vector containing the murine PAH cDNA	Systemic delivery	• Complete correction of HPA in both males and females with a rAAV8 vector	Division of Clinical Chemistry and Biochemistry, University Children’s Hospital Zürich	Gene Ther. 2006 April; 13 (7):587–93
			• Reversion of hypopigmented phenotype		
ssAAV8/CAG-mPAH and scAAV/LP1-mPAH	An AAV8-pseudotyped vector constructed with a self-complementary AAV (scAAV) genome	Systemic delivery	• Long duration of the treatment	Division of Genetic Therapeutics, Center for Molecular Medicine, Jichi Medical University, Shimotsuke, Japan	J Gene Med. 2011 February; 13 (2):114–22
			• Reduction of blood Phe to normal or near-normal levels for more than 1 year in both genders		
AAV8-AAVR and the SaCas9 system	Co-delivery of SaCas9/sgRNA/donor templates with AAVR *via* AAV8 vectors	Systemic delivery	• Dramatically increased AAV transduction efficiency *in vitro* and *in vivo*	Shanghai Key Laboratory of Regulatory Biology, Institute of Biomedical Sciences and School of Life Sciences, East China Normal University, Shanghai, 200241, China	Sci China Life Sci. 2022 April; 65 (4):718–730
			• Increased indel rate over 2-fold and homologous recombination rate over 15-fold for the correction of the single mutation in PahR408W mice		
			• Significantly decreased Phe level and ameliorated PKU symptom		
rAAV2/8-hPAH	A pseudotyped recombinant AAV2/8-hPAH vector and infused it into female PKU mice through the hepatic portal vein or tail vein	Systemic delivery	• Increased PAH activity (female: 65–70%; male: 90%)	Department of Biochemistry, School of Medicine, Ewha Womans University, Seoul, Korea	J Korean Med Sci. 2008 October; 23 (5):877–83
			• Plasma Phe concentration in female decreased to the normal value		
			• The offspring of the treated female PKU mice can rescue from the harmful effect of maternal HPA		
			• Reversion of hypopigmented phenotype		
rAAV2/1-PAH-GTPCH-PTPS	A rAAV2 pseudotype 1 vector expressing PAH along with GTPCH and 6-pyruvoyltetrahydrobiopterin synthase (PTPS)	Gastrocnemius muscles injection	• The stable and long-term reduction of blood Phe	Division of Clinical Chemistry and Biochemistry, Department of Pediatrics, University of Zürich	Mol Ther. 2008 April; 16 (4):673–81
			• Reversal of PKU-associated coat hypopigmentation		
AAV2/1-PKU5, AAV2/2-PKU5, and AAV2/8-PKU5	Three different recombinant AAV2 genomes packaged with either serotype 1, 2, or 8 capsid were generated to express the PAH gene	Gastrocnemius muscles injection	• Restored liver PAH activity and Phe clearance (long-term clearance: AAV2/1-PKU5 and AAV2/8-PKU5 only)	Division of Clinical Chemistry and Biochemistry, Department of Pediatrics, University of Zürich, CH-8032 Zürich, Switzerland	Hum Gene Ther. 2010 April; 21 (4):463–77
			• Therapeutic correction in both genders (more effectively in males)		
			• Subsequent supplementation with synthetic tetrahydrobiopterin resulted in a transient decrease in blood phenylalanine in female after rAAV2/8 injection		
AAV5/CAG-mPAH.	A recombinant adeno-associated virus (AAV) vector carrying the murine PAH cDNA	Systemic delivery	• Completely normalized pf PHA phenotype	Division of Genetic Therapeutics, Center for Molecular Medicine, Jichi Medical School, Tochigi, Japan	Gene Ther. 2004 July; 11 (13):1081–6
			• Substantial blood Phe reduction in male		
			• Long-term correction of HPA		
			• Transduction ameliorated the PKU phenotype (reversed central nervous system dysfunctions and correction of hypopigmentation)		
rAAV-mPAH-WPRE	A rAAV-mouse phenylalanine hydroxylase-woodchuck hepatitis virus post-transcriptional response element (rAAV-mPAH-WPRE) vector	Systemic delivery	• Rapid reduction of serum Phe levels	Department of Biochemistry and Molecular Biology, PO Box 100245, College of Medicine, University of Florida, Gainesville, FL 32610, United States.	Brain Res. 2007 January 5; 1127 (1):136–50
			• Reversed neuropathologic phenotypes		
AAV8-miniSaCBE-PLUS-PKU	A dual AAV strategy for *in vivo* delivery of base editors, in which deaminases were linked to Cas9 through the interaction of GCN4 peptide and its single chain variable fragment (scFv) antibody	Systemic delivery	• Up to 27.7% correction *in vitro*	Laboratory of Biotherapy, National Key Laboratory of Biotherapy, Cancer Center, West China Hospital, Sichuan University, Renmin Nanlu 17, Chengdu 610041, Sichuan, China	Mol Ther Methods Clin Dev. 2022 Jan 7; 24: 230–240
			• Significantly rescued Phe metabolism and reduced urine phenyl ketone		
			• Rescue of hyperphenylalaninemia-associated syndromes *in vivo* (e.g. growth retardation, hypopigmentation, and behaviors)		
AAVHSC15-PAH	AAVHSC15: a clade F AAV isolated from human CD34^+^ hematopoietic stem cells (HSCs) AAVHSC15-PAH: The AAVHSC15 vector containing a codon-optimized form of the human phenylalanine hydroxylase cDNA	Systemic delivery	• Sustained reduction of serum Phe	Research and Development, Homology Medicines, Patriots Park, Bedford, MA 01730, United States.	Mol Ther Methods Clin Dev. 2020 March 13; 17:568–580
			• Sustained reduction in serum Phe and normalized tyrosine levels for the lifespan of Pah^enu2^ mice		
			• Restored brain levels of Phe and the downstream serotonin metabolite 5-hydroxyindoleacetic acid		
			• Reversal of PKU-associated coat hypopigmentation		

An ideal strategy for gene therapy is expected to be non-pathogenic, transduction efficient, as well as sustained long-term expressed. PAH-deficient mice achieved nearly complete restoration of liver PAH activity and reversed symptoms without dietary supervision using AAV-mediated gene therapy. Despite the conventional AAV-pseudotype vectors, a series of newly developed AAV vectors also achieved the satisfactory results. Harmful changes in the brains of Pah^enu2^ mice were reversed after the portal vein delivery of an rAAV-mouse PAH-woodchuck hepatitis virus post-transcriptional response element (rAAV-mPAH-WPRE) vector (Embury et al., 2007). Kaiser et al. (2021) synthesized the AAV vector Anc80, a synthetic serotype using *in silico* techniques, to deliver a functional copy of a codon-optimized human PAH gene to the Pah^enu2^ mice. They observed the circulating Phe was reduced nearly to the control levels in males, but the clinic curative effect still needs to be proved in human trials.

## Conclusion

PKU results from the severe form of HPA, a syndrome recognized by high concentrations of blood Phe. Consequently, blood Phe levels is the most important marker for diagnosis and treatment. Over the past 50 years, the classical lifelong dietary treatment has been regarded as the most effective approach to prevent disease consequences. Recently, the pharmaceutical therapies have provided alternative options for treatment. Several studies have suggested that AAV-based gene therapy might be another promising approach for HPA curation using only one-time administration without dietary restriction, while its security and efficacy await the results of ongoing clinical trials. Considering the uncertainties around the capacity and long-term durability of gene therapy, more optimizations are needed in the near future.
